# Health systems resilience in fragile and shock-prone settings through the prism of gender equity and justice: implications for research, policy and practice

**DOI:** 10.1186/s13031-022-00439-z

**Published:** 2022-02-21

**Authors:** Wesam Mansour, Abriti Arjyal, Chad Hughes, Emma Tiange Gbaoh, Fouad Mohamed Fouad, Haja Wurie, Hnin Kalayar Kyaw, Julie Tartaggia, Kate Hawkins, Kyu Kyu Than, Lansana Hassim Kallon, Maya Abou Saad, Obindra Chand, Phone Myint Win, Rouham Yamout, Shophika Regmi, Sushil Baral, Sally Theobald, Joanna Raven

**Affiliations:** 1 Department of International Public Health, Liverpool School of Tropical Medicine, Pembroke Place, Liverpool, L3 5QA UK; 2 HERD International, Kathmandu, Nepal; 3 Burnet Institute, Melbourne, Australia; 4 College of Medicine and Allied Health Sciences, University of Sierra Leone, Freetown, Sierra Leone; 5 Faculty of Health and Sciences, American University in Beirut, Beirut, Lebanon; 6 Burnet Institute, Yangon, Myanmar; 7 Pamoja Communications Ltd., Brighton, UK

**Keywords:** Gender, Equity, Health systems, Resilience, Fragile and shock-prone settings

## Abstract

Fragile and shock-prone settings (FASP) present a critical development challenge, eroding efforts to build healthy, sustainable and equitable societies. Power relations and inequities experienced by people because of social markers, e.g., gender, age, education, ethnicity, and race, intersect leading to poverty and associated health challenges. Concurrent to the growing body of literature exploring the impact of these intersecting axes of inequity in FASP settings, there is a need to identify actions promoting gender, equity, and justice (GEJ). Gender norms that emphasise toxic masculinity, patriarchy, societal control over women and lack of justice are unfortunately common throughout the world and are exacerbated in FASP settings. It is critical that health policies in FASP settings consider GEJ and include strategies that promote progressive changes in power relationships. ReBUILD for Resilience (ReBUILD) focuses on health systems resilience in FASP settings and is underpinned by a conceptual framework that is grounded in a broader view of health systems as complex adaptive systems. The framework identifies links between different capacities and enables identification of feedback loops which can drive or inhibit the emergence and implementation of resilient approaches. We applied the framework to four different country case studies (Lebanon, Myanmar, Nepal and Sierra Leone) to illustrate how it can be inclusive of GEJ concerns, to inform future research and support context responsive recommendations to build equitable and inclusive health systems in FASP settings.

## Introduction

Fragility, violence, conflict, and other shocks present a critical development challenge, eroding efforts to build healthy, equitable, and inclusive societies. In Fragile and Shock-Prone (FASP) settings, innovative, timely and contextually tailored evidence generated in partnership with local and national stakeholders is critical to developing and sustaining equitable and resilient health systems. Power relations experienced by people because of social markers, e.g., gender, disability, education, age, ethnicity and race, can intersect leading to poverty, marginalisation, and associated health challenges, increasing inequities. Intersectionality and its application are gaining traction in global health [1]. It is an approach that supports analysis of how policies can promote gender, equity, and justice (GEJ), and strategies to ensure ‘no one is left behind’ in the attainment of the Sustainable Development Goals [2]. The concept of ‘intersectionality’ was coined by black American activist, Kimberlé Crenshaw in 1989 as a lens to explain how various forms of inequality can operate together simultaneously, exacerbating each other and intersecting to create different modes of discrimination and privilege [3]. An increased focus on intersectionality has highlighted gaps, for example, in the design of gender-based violence (GBV) programmes. Guidance documents on GBV are often limited in their focus on vulnerable groups, reinforcing the idea of separate and innate identities, rather than overlapping social positions inevitably linked with structures of oppression [4]. There is a growing body of literature that explores the impact of these intersecting axes of inequity in FASP settings, but gaps remain when it comes to what actions can promote GEJ.

Gender norms are social rules that define the behaviour of people of any gender or age in any given society and restrict their gender identity into what is considered to be appropriate [5]. Gender norms that emphasise toxic masculinity and societal control over women occur throughout the world and are exacerbated in FASP settings [6]. Patriarchal control over women limits their role in household decision-making and acts as a precursor for sexual and gender-based violence (SGBV) during conflicts [7]. Lesbian, gay, bisexual, transgender and queer or questioning (LGBTQ+) people are also among the most discriminated, encountering verbal, mental and sexual violence especially during conflicts [8].

The COVID-19 pandemic has further exacerbated gender norms that prescribe caregiving roles, for example, with women being more at risk of infection, both as domestic caregivers and healthcare workers [9]. School closures and travel restrictions limited women’s work and economic opportunities since in many contexts, gender norms prescribed that they needed to stay home and take care of their children during the pandemic time as primary domestic caregivers [10]. Refugee girls are only half as likely to enrol in secondary-level education as their male peers, with inequities exacerbated by school closures [11]. In conflicts, many men die, leaving behind female-headed households. During extreme hardship, these households often lack financial resources, which might affect their children who may engage in child labour or early marriage to alleviate the financial burden [12]. These gender norms and power relations play a significant role in determining access to and the outcomes of health services [13]. For example, access to sexual and reproductive health services, including contraception, is often limited, leading to unplanned pregnancies, more children leading to protracted poverty [14]. Elderly people and people with disabilities (PWDs) are often not considered when designing interventions and planning service provision, and FASP settings can exacerbate their vulnerabilities [15], leading to mental health challenges [16]. Discrimination against PWDs can extend to their families, leading to isolation and exclusion from services, aggravated by poor communication systems which restrict access to information and healthcare services.

To improve health outcomes and service delivery to diverse vulnerable populations and marginalised individuals and groups, it is critical that health policies at all levels consider GEJ and include strategies that allow effective implementation and foster progressive changes in power relationships among people of all genders [17]. These can be mechanisms to promote positive gender norms, roles and behaviours [18]. Disease outbreak responses can provide an important catalyst for work on GEJ within preparedness and response efforts—improving the effectiveness of health interventions and promoting GEJ [17].

**Fig. 1:**
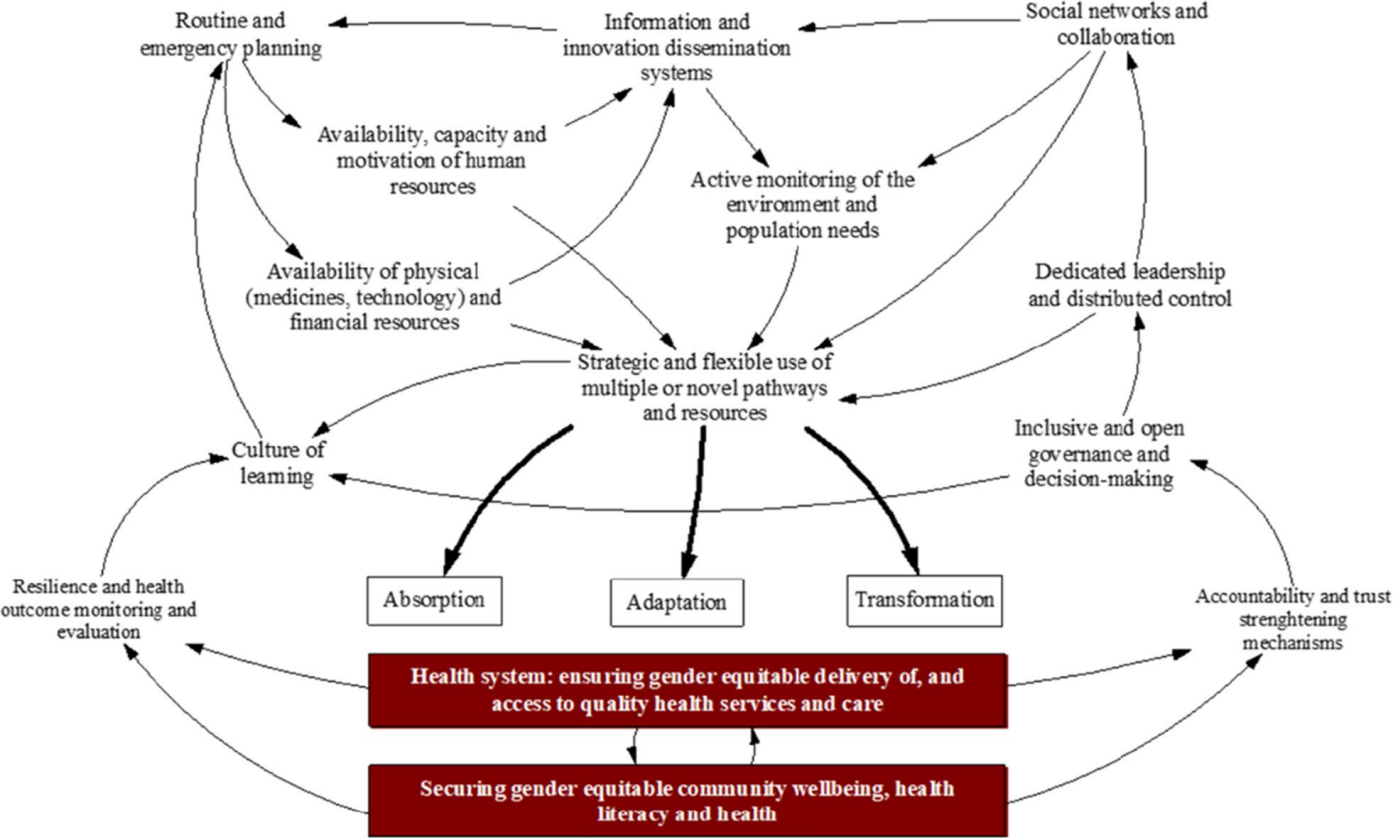
ReBUILD for resilience framework ^19^

ReBUILD for Resilience (ReBUILD) is a multi-country health systems research consortium (https://www.rebuildconsortium.com/). Our work focuses on resilience in Lebanon, Myanmar, Nepal and Sierra Leone and is underpinned by a conceptual framework that is grounded in a broader view of health systems as complex adaptive systems (Fig. 1) [19]. Our outcomes have gender and equity at their heart (as illustrated by the red rectangles).

Each ReBUILD country has faced shocks that impacted its health system, and each continues to face challenges brought by COVID-19. The ReBUILD GEJ Working Group held a set of webinars to initiate a dialogue across the consortium and beyond to explore our conceptualisation of GEJ in FASP settings. Different organisations and international consultants with expertise in GEJ participated in these discussions and provided empirical examples to promote GEJ in health system research. For the purposes of this paper, we gathered contextual evidence and tacit knowledge within our evolving settings to understand how the GEJ concepts link to our resilience framework. To inform our analysis and to structure this paper, we clustered the resilience framework nodes around three cross-cutting areas: governance, decision-making and accountability; human resources for health; and learning, monitoring and evaluation. For each area, we mapped the implications for GEJ research, policy and practice, including illustrative examples from ReBUILD. Our learning is synthesised here with priority research areas and ways forward for each of the three cross cutting areas highlighted to inform our ReBUILD work and also support policymakers, governmental officials at all levels, academics and health system researchers in FASP settings and beyond to support building equitable and inclusive health systems within these fragile settings.

## Inclusion, open governance, decision-making and accountability

The politics of intervention implementation, the diverse actors involved, and contextually specific power structures can influence service provision at various levels of health systems in FASP settings. Improving accountability and decision-making processes for engaging communities in health service planning is important.

Nepal has a new federal structure of the health system functioning at three tiers—federal, provincial, and local levels. With federalism came a considerable shift in Nepal’s governance mechanism, which is now more inclusive with decentralised decision-making. Local governance structures mandated the representation of marginalised groups including women and disadvantaged/minorities in various positions/committees. These representatives are responsible for executive and judicial functions and with this comes greater power. However, there is little meaningful participation from marginalised groups, with men taking most decision-making roles. Multiple GEJ responsive strategies were developed in the health sector [20, 21], partially driven by the 2015 earthquake and other environmental and economic shocks that highlighted the neglect of marginalised groups [21]. However, mainstreaming strategies into the institutional and implementation frameworks and ensuring government capacity to deliver them is a challenge.

Since Myanmar’s independence in 1948, armed conflicts between Myanmar military and Ethnic Armed Organizations (EAOs) have contributed to a fractured health system. Prior to the military deposing the existing government and assuming power in February 2021, Myanmar supported parallel health systems: government-run services in government-controlled areas under the National Health Plan (NHP), and those run by Ethnic Health Organisations (EHOs) serving ethnic minorities in conflict-affected areas. Women’s participation in decision-making in public and political life, including peace processes, has received growing attention in recent years. Following the 2015 election, where Aung San Suu Kyi became de facto leader of the country, the percentage of women in Myanmar’s Hluttaw (council of ministers) more than doubled to 10.5%, but is still below the Asian average (19.4%) and global average (23.3%) [22]. Since the 2021 military coup, most services in all areas of the country are disrupted [23]. During the 2010 shift to a military-civilian government, the NHP outlined a path towards decentralisation with minimal involvement of EHOs, but progress was slow, perhaps due to the overall weakness of the health system including inadequate managerial capacity [24]. Integrating goals and actions to address GEJ issues into the NHP and indicators that allow for more robust gender and intersectional analysis, will build accountability for GEJ-considerate decision-making at all health system levels [24]. Another priority step is to build on existing GEJ supportive mechanisms/approaches, including formalising permanent seats in health decision-making bodies at all levels for civil-society organisations representing women and marginalised groups, which is currently an ad-hoc informal approach.

In Lebanon, health governance often works in favour of the political elite, representatives of sectarian political powers and powerful lobbies (private hospitals, physicians, pharmaceutical companies) rather than in the engagement of health policies in providing health for all [25]. This results in inappropriate, poorly reinforced, inapplicable decisions, and greater injustice in access of vulnerable populations to affordable and quality healthcare. Key priorities include restructuring the decision-making process, and implementing new ways of evidence-based, decentralised, equitable and population-centred healthcare governance that is informed by research. Channelling aid to Syrian refugees through public institutions also faces challenges of lack of transparency, sectarianism and clientelism [26]. The international response is complex and some of this is disconnected from the local context, with limited transparency in financial transactions [26], leading to untrusted and inequitable services. Accountability and transparency in auditing, financial reporting and budgeting are needed across all health actors [26].

### Actions and way forward

The ReBUILD learning and discussions initiated across our consortium and beyond, focused on approaches that could promote inclusion, open governance, decision-making and accountability for GEJ in health systems, and the potential opportunities presented by decentralisation (as highlighted by the Nepalese context) Moving forward in ReBUILD we will work collaboratively in a”Learning sites” approach which will provide an opportunity to explore decision-making and governance at the local level [27]—working with local stakeholders to map what is happening and identifying required actions, e.g., making decision-making more gender equitable and inclusive, ensuring marginalised groups are heard, and strengthening accountability mechanisms. These actions can be implemented, documenting the innovations, reflections and adaptations over time, taking note of what changes and why, what is blocked, and what facilitates action, innovation, and equitable processes.

Community engagement, with the meaningful participation of different groups in planning decision-making and execution is important. For example, in ReBUILD we will move this forward by evaluating community feedback mechanisms for greater accountability, improved quality, increased trust in the health system, and working with community health workers (CHWs) to support their engagement in decision-making at the community level. Health systems that have open and inclusive governance structures, where local leadership and distributed control of resources are emphasised, are likely to be more efficient in implementing, testing and revising adaptive solutions for service delivery in times of shock. We will also explore how gender and leadership can be strengthened at the local level.

## Responsive human resources for health policies for gender, equity and justice

In FASP settings, there is an urgent need to understand how human resources (HR) management can contribute to health systems rebuilding. However, rebuilding with competent and equitably distributed workforce is often complex [28]. In Lebanon, HR challenges are exacerbated by the severe economic crisis since 2019, followed by COVID-19, leading to a dramatic decline in health workers’ income, especially among already underpaid non-physician workers [29]. Health facilities which have not received payment from public insurance schemes [30] resorted to cutting salaries and dismissing personnel [31], leading to an exodus of physicians [32]. This situation negatively impacts on the quality [33], appropriateness [34] and availability [35] of healthcare services and jeopardises the COVID-19 response, depriving the most vulnerable of testing and treatment. A lack of female healthcare providers was a challenge faced by female Syrian refugees [36]. This may become more of a problem as 80% of nurses are women [37] and are facing greater lay-offs and wage reductions than their male counterparts [38]. The informal health sector emerged as a short-term solution to these gaps [39], but workers need to be integrated into the national health system, with payment, regulation, training and support provided.

HR challenges are a perennial problem for Nepal’s health system [40], and have continued with the introduction of the new federal structure. The chronic shortage of staff, problems deploying health workers to rural areas, and limited benefits and motivation led to absenteeism which influenced service provision and imposed further challenges to HR management during COVID-19 [41]. With an expanding urban poor and changing health needs, the health workforce lacks the capacity to address populations’ needs. Although 40% of the health workforce in Nepal are women, most are not in leadership positions [42]. These issues can be addressed by supporting HR at all levels through strengthening capacity, enabling women leaders, and creating reward systems to motivate health workers to provide services in hard-to-reach areas.

In Myanmar, the 2021 military coup risks reversing recent gains in supporting a gender equitable health workforce. Medical personnel were among the first to join the civil disobedience movement and have since been under threat of arrest. This frontline role taken by healthcare workers, and concerns for their safety, limits their capacity to provide healthcare, including free services to the public outside formal structures. This led to a severe shortage of health staff and serious challenges in maintaining basic health services, compounded by COVID-19.. Before the coup, university entry and opportunities for female students were equal to males, and educational reform was a national priority, supporting health workforce education and training. However, career pathway into policy making roles is less equitable: men and women doctors appear to have equal opportunities, but nurses, where the majority are women, have fewer opportunities. Since the coup, most universities have closed, and reform plans have halted [43]. Once the country stabilises and educational reform is again on the agenda, a culture of learning in relation to GEJ issues for medical, public and allied health students could be nurtured by embedding critical thinking, decision-making and problem-solving skills into education.

In Sierra Leone, the health system is characterised by inequitable and inefficient allocation of HR [44], which is further exacerbated by shocks. During the Ebola outbreak, frontline health workers were mostly from lower cadres and women (out of 11,325 deaths, 59–75% were women, including health workers) [45]. In the initial phase of the Ebola response, there were insufficient infection prevention and control materials [46], which increased the infection rate, putting women at risk. CHWs took on critical roles during the Ebola response and in the COVID-19 response including education about safety, contact tracing and visiting those quarantined. Societal and gender norms continue to play out, with female CHWs facing challenges travelling around the community due to the nature of the terrain and mode of transportation available, female supervisors not being recognised by male supervisees, the dual burden of community and household work, including home schooling during lockdown. This limits their ability to engage in other income generating activities, creating challenges for female-headed households. Infection mitigation measures such as curfews and lockdowns (including inter-district lockdowns) created significant threats to female-run micro-level businesses e.g., street hawking including food vendors, market traders, retailing and domestic services, drastically impacting on women's livelihoods and economic security with wider implications for food security. CHWs’ workloads during COVID-19 prevented them from engaging in other income generation exercises, further decreasing their earning power, and government incentives were irregular [47].

### Actions and way forward

We identified several ways that HR policy and practice could be improved, so that health systems in FASP contexts can better support health workers in their critical roles and enable them to be more responsive to GEJ concerns. Including people of all genders and backgrounds in the health workforce would help develop GEJ responsive and transformative HR. Policy examples include recruitment and retention, training and career development policies that promote equal opportunities and counter discrimination. These policies need to be disseminated and operationalised by sub-national level managers who can adapt these polices so that they respond to the local contexts. Accurate information about the numbers and distribution of staff in rural and urban areas can inform decision-making so that there is equitable deployment of staff, reflecting specific contextual needs. For example, in ReBUILD, we will promote the availability and use of gender and equity disaggregated data on HR in learning sites to inform actions and develop evidence on CHWs’ gendered experiences and needs related to providing services in the context of fragility, COVID-19, and future shocks.

## A culture of learning, monitoring and evaluation for gender, equity, and justice

In learning health systems, evidence and data is used routinely to inform day-to-day decision-making and to respond to shocks. In our settings, there is a need for strong accountability mechanisms, informing how routine data systems can capture equity concerns and inform decision-making. Monitoring and evaluation of resilience depend on appropriate metrics and processes that are used to appraise health system resilience, including measures of preparedness, responsiveness and recovery, and how these are operationalised in FASP settings.

Sierra Leone’s health system has dealt with multiple shocks, e.g., civil war, Ebola and COVID-19. The epidemics led to the establishment of the Emergency Operation Centre to lead the responses. Despite the government’s effort to increase women representation in leadership and decision-making spaces by 30%, currently leadership and decision-making roles are male dominated, and GEJ considerations are often overlooked. This was evident in the recent Ebola outbreak response which failed to adequately plan for vulnerable groups, leading to decreases in service use and worsening outcomes for pregnant women, lactating mothers, under-fives and PWDs due to the fear of infection [44, 48]. The response also overlooked women and girls’ caregiving roles, and the added risk they face during outbreaks [48, 49]. Many of the Ebola burial teams were made up of young men, and because of limited psychosocial support, their mental wellbeing was affected [50], which can have long-term implications. The post-Ebola recovery plan considered gender and social protection [48], but this has not shaped ongoing practice or a culture of change and the subsequent national health security plan (2018–2020) appears to lack GEJ considerations. The translation of gender transformative policies was influenced by gender and societal norms, resulting in unintended consequences. For example, the CHW policy developed after the Ebola was design to recruit more female CHWs but in practice more male CHWs were recruited. National measures are being put in place to address these norms, including some policies and laws to promote GEJ. However, a more consultative approach should be considered to ensure intended implementation, ensuring stakeholders and social structures are engaged to gradually engender change.

In Nepal, limited indicators in the Health Management Information System (HMIS) restrict robust gender and intersectionality analysis and impede the planning of inclusive health services. There is a need to strengthen the HMIS to incorporate wider social stratifiers, disaggregated analysis and data use in local decision-making, with stakeholder capacity development at different levels.

### Actions and way forward

Our discussions highlight how investments in monitoring and evaluation are limited compared to other health systems areas, in addition to poor use of routine data for equity analysis and driving change. There is need to invest in developing health indicators to assess equity from an intersectional standpoint (e.g., by gender, age, dis/ability), and to use this disaggregated data to assess equity in ways that are usable, acceptable, relevant and actionable at different levels, including within community health. This and other relevant data (including from qualitative and participatory research processes) should inform and strengthen a culture of embedded and responsive M& E within annual health systems review processes and building decision-making space to actively address local level inequities. This requires training and building networks for change to inspire action. We noted the opportunity that learning sites present local health systems actors to identify areas for improvement, innovate for action and learn from the process, thus embedding a culture of reflection and learning into the system. Dissemination of GEJ information (i.e., policy briefs and case studies) that can be used by policymakers and implementers is important. Identifying and investing in innovative and culturally appropriate techniques (e.g., using local languages and different delivery mediums) to communicate messages to marginalised group will be key. There is a need to strengthen the political will and demand for monitoring, evaluation and learning systems that promote equity and inclusiveness. This may include nurturing change champions and trusted institutions to promote GEJ within health systems, while building accountability and responsiveness.

## Conclusion

The ReBUILD for Resilience framework identifies links between different capacities and enables identification of feedback loops which can drive or inhibit the emergence and implementation of resilient approaches. The framework acknowledges that resilience is not an end in itself, but a step towards securing equitable community wellbeing, health literacy and health. We applied it to four different case studies to illustrate how it can be inclusive of GEJ concerns, to inform future research and support contextually sensitive recommendations to build equitable and inclusive health systems in FASP settings. Moving forward a learning sites approach will help explore decision-making and governance at the local level and promote the availability and use of gender and equity disaggregated data on HR to inform actions and develop the evidence base on health workers’ gendered experiences and needs in FASP settings.

## Data Availability

Not applicable.

## Abbreviations

CHWs: Community health workers
EAOs: Ethnic Armed Organizations
EHOs: Ethnic Health Organizations
FASP: Fragile and shock-prone
GEJ: Gender, equity and justice
HMIS: Health Management Information System
HR: Human resources
PWDs: People with disabilities
SGBV: Sexual and gender-based violence
